# Increased MicroRNA-34b and -34c Predominantly Expressed in Stromal Tissues Is Associated with Poor Prognosis in Human Colon Cancer

**DOI:** 10.1371/journal.pone.0124899

**Published:** 2015-04-20

**Authors:** Yukiharu Hiyoshi, Aaron J. Schetter, Hirokazu Okayama, Kentaro Inamura, Katsuhiro Anami, Giang H. Nguyen, Izumi Horikawa, Jason E. Hawkes, Elise D. Bowman, Suet Yi Leung, Curtis C. Harris

**Affiliations:** Laboratory of Human Carcinogenesis, Center for Cancer Research, National Cancer Institute, National Institutes of Health, Bethesda, Maryland; Rutgers, the State University of New Jersey, UNITED STATES

## Abstract

The microRNA-34 family (miR-34a, -34b and -34c) have been reported to be tumor suppressor microRNAs (miRNAs) that are regulated by the TP53 and DNA hypermethylation. However, the expression, regulation, and prognostic value of the miR-34 family have not been systematically studied in colon cancer. To elucidate the roles of miR-34 family in colon carcinogenesis, miR-34a/b/c were measured in tumors and adjacent noncancerous tissues from 159 American and 113 Chinese colon cancer patients using quantitative RT-PCR, and we examined associations between miR-34a/b/c expression with TNM staging, cancer-specific mortality, TP53 mutation status and Affymetrix microarray data. All miR-34 family members were significantly increased in colon tumors, counter to the proposed tumor suppressor role for these miRNAs. Increased miR-34b/c were observed in more advanced tumors in two independent cohorts and increased expression of miR-34b/c was associated with poor cancer-specific mortality. While the expression of miR-34 family was not associated with TP53 mutation status, TP53 transcriptional activity was associated with miR-34a/b/c expression that is consistent with the proposed regulation of miR-34a/b/c by TP53. To examine where the miR-34 family is expressed, the expression of miR-34 family was compared between epitheliums and stromal tissues using laser microdissection technique. The expression of miR-34b/c was increased significantly in stromal tissues, especially in cancer stroma, compared with epithelial tissue. In conclusion, increased miR-34b/c predominantly expressed in stromal tissues is associated with poor prognosis in colon cancer. MiR-34 may contribute to cancer-stromal interaction associated with colon cancer progression.

## Introduction

Colorectal cancer is the third most common cancer and the fourth leading cause of cancer-related death worldwide. Approximately 1.2 million new cases and about 600,000 deaths estimated occur annually [1]. Further studies to discover risk factors, biomarkers and therapeutic targets for colorectal cancer may help reduce the burden of this cancer.

MicroRNAs (miRNAs) are non-coding, single-stranded RNAs that are 18–25 nucleotides long. Since their discovery, miRNAs have been found to down-regulate the expression of multiple genes by interacting with the 3’UTR of target mRNAs to signal their degradation and/or blocking their translation into proteins [2]. Furthermore, these molecules regulate genes involved in a variety of cellular processes such as apoptosis, differentiation, proliferation, stress response and metabolism, and their deregulation or dysfunction plays an important role in carcinogenesis and tumor progression [3–5]. Specific miRNA expression signatures have been found in many malignancies, including colorectal cancer [6–8].

The miR-34 family is largely considered to be a tumor suppressor miRNA [9]. This family consists of three members: miR-34a, generated from a transcriptional unit on the human chromosome 1p36, and miR-34b and miR-34c, which are generated by processing of a bicistronic transcript from chromosome 11q23. Both transcripts have been shown to be under the direct positive control of p53 [10–12]. Several converging evidence demonstrated that ectopic expression of miR-34 family induces apoptosis, senescence, cell cycle arrest and inhibits migration and invasion [9, 13]. Therefore, miR-34 family is thought to be important mediator of p53’s tumor-suppressive activities. Besides, the miR-34a and miR-34b/c genes display varying levels of DNA methylation in numerous types of tumors including colorectal cancer, and may therefore represent tumor suppressor genes [14–16]. Although previous reports have demonstrated that down-regulation of miR-34 family members was associated with poor prognosis in several types of malignancies [17–20], the prognostic value of miR-34 family in colon cancer has not been systematically studied.

To evaluate the issue, we set out to systematically study the expression of the miR-34 family in two large, independent cohorts of colon cancer patients. In addition, the association between miR-34 expression and TP53 mutation, global gene expression was examined to clarify possible mechanisms of miR-34 regulation. To our knowledge, this is the largest and systematic study analyzing miR-34 expression in human colon cancer.

## Material and Methods

### Clinical samples

Pairs of primary colon tumor and adjacent non-tumor tissues came from 159 patients recruited from the University of Maryland Medical Center or Baltimore Veterans Affairs Medical Center between 1993 and 2002, and from 113 patients recruited from Queen Mary Hospital in Hong Kong between 1991 and 2000. Detailed backgrounds for each tissue donor, including age, sex, clinical staging, tumor location and survival times from diagnosis have been collected. The final date of follow-up was December 31, 2008 for the American cohort and August 16, 2004, for the Chinese cohort. Tumor histopathology was classified according to the World Health Organization Classification of Tumor system.

### Ethics Statement

This study was approved by the Institutional Review Board of the National Institutes of Health, the Institutional Review Board of the University of Hong Kong/Hospital Authority Hong Kong West Cluster, and the Institutional Review Board for Human Subject Research at the University of Maryland. All participants provided their written informed consent to participate in this study, and the consent procedure was approved by the Institutional Review Board.

### RNA isolation and quantitative reverse transcriptase-PCR

RNA from frozen tissue samples and cell lines was extracted using standard TRIZOL (Invitrogen, Carlsbad, California) methods. RNA was reverse-transcribed using TaqMan MicroRNA Reverse Transcription Kit for miRNA assay (Applied Biosystems, Foster City, California). Quantitative-PCR of microRNAs was performed using TaqMan assays (Applied Biosystems, Foster City, California) according to the manufacturer’s instructions with the 7900 HT Real-Time RT-PCR System (Applied Biosystems, Foster City). All assays were performed in triplicate. All TaqMan probes were purchased from Applied Biosystems; hsa-miR-34a-5p (Assay ID: 000426), hsa-miR-34b-5p (ID: 000427), hsa-miR-34c-5p (ID: 000428), RNU66 (ID: 001002). All microRNAs were normalized to RNU66 using the -ΔCt method. MicroRNA expressions were further normalized against the average expression of nontumor for each microRNA for each cohort separately with the following equation, [-ΔCt_(average nontumor)_]–[-ΔCt_(sample)_]. This sets the average nontumor expression = 1, with the log2 nontumor expression = 0.

### Affymetrix mRNA array and Ingenuity Pathway Analysis (IPA)

RNA from 56 tumors from the American cohort were analyzed on Affymetrix U133A mRNA microarrays. Array data was imported and RMA normalized using Partek Genomic Suites 6.6. Data is publically available at (GSE44861). The average expression of miR-34b and miR-34c was used to analyze the association of miR-34b/c expression and global mRNA expression. Ingenuity Pathway Analysis (IPA 8.0, Redwood city, CA) was performed to identify molecules and pathways potentially involved with the expression of miR-34 family.

### Laser microdissection

For Laser microdissection (LMD), 5 pairs of frozen tumor and non-tumor tissues were randomly obtained from the American cohort. Seven micron frozen sections for each tissues were placed on the PEN membrane slides (Leica Microsystems). Sections were fixed in 75% ethanol for 1 min, stained with Nuclear fast red for 2min, and dehydrated in a graded series of ethanol and xylen. Slides were air-dried and then microdissected using the LMD6000 system (Leica Microsystems). Microdissected epithelial and stromal cells from tumor and non-tumor tissues were collected directly into Qiazol, and total RNA was isolated using the RNeasy Micro kit (Qiagen) according to the manufacturer’s instructions. qRT-PCR was performed as described earlier.

### Statistical analysis

Relative expression quantitation of microRNA was calculated with RQ manager 1.2 (Applied Biosystems). Expression values were used to analyze differences in microRNA expression between tumors and adjacent non-tumor tissues for all qRT-PCR data using Graphpad Prism v5.0 (Graphpad Software Inc, San Diego, California). Wilcoxon matched-pairs tests were used to compare microRNA expression between tumors and paired nontumor tissues. All trend tests reported are Cuzick nonparamtric test for trend across ordered groups [21] and were performed using Stata 9.2 (StataCorp LP, College Station, Texas). Kaplan-Meier analysis was performed using Graphpad Prism v5.0. All p values were 2-sided and statistical significance was set at p<0.05.

## Results

This study used two independent cohorts of colon cancer patients, one consisting of 159 cases recruited from Maryland and the second cohort of 113 cases recruited from Hong Kong, China (Table 1). The median follow-up times were 90.3 and 84.6 months for patients in the American and the Chinese cohorts, respectively. The cohorts were similar in TNM staging, tumor histology and cancer-specific mortality. The 5-year survival rate was 46.4% for the American cohort and 49.6% for the Chinese cohort and were not significantly different from one another (P = 0.36, Kaplan-Meier test). In addition to the racial, geographic and cultural differences between these 2 cohorts, the American cohort was considerably older (average 66.2 years vs 55.8 years) had a higher percentage of men (71% vs 50%). The Chinese cohort had a higher percentage of tumors located at distal colon (80% vs 20%). TP53 mutation evaluated by immunohistochemistry was detected in 50.6% of the American cohort and 51.8% of the Chinese cohort.

**Table 1:** Characteristics of study populations and tumors.

	American cohort	Chinese cohort	P-value
	n = 159	n = 113	
Area of recruitment	Baltimore, MD	Hong Kong, China	
Age at enrollment (y)			
Mean (SD)	66.2 (11.3)	55.8 (14.9)	P<0.0001
Range	32–87	32–84	
Gender, no. (%)			P = 0.0003
Male	113 (71.1)	56 (49.6)	
Female	46 (28.9)	57 (50.4)	
Tumor location^a^, no. (%)			P = 0.0014
Distal	48 (58.5)	90 (79.6)	
Proximal	34 (41.5)	23 (20.4)	
Adenocarcinoma histology, no. (%)			NS
Adenocarcinoma	142 (89.3)	105 (92.9)	
Mucinous adenocarcinoma	16 (10.1)	7 (6.2)	
Adenosquamous	1 (0.6)	0 (0)	
Signet ring cell and mucinous	0 (0)	1 (0.8)	
TP53 status^b^, no. (%)			NS
Wild-type TP53	39 (49.4)	52 (48.2)	
Mutant TP53	40 (50.6)	56 (51.8)	
TNM staging^c^, no. (%)			NS
I	21 (13.3)	9 (8.0)	
II	54 (34.2)	37 (32.7)	
III	65 (41.1)	48 (42.5)	
IV	18 (11.4)	19 (16.8)	

^a^ Distal includes tumors located in or distal to the descending colon. Proximal tumors include tumors in or proximal to the splenic flexure. Tumor location was available for all cases in the Chinese cohort and 82 cases in the American cohort.

^b^ TP53 status was evaluated by immunohistochemistry of Formalin-Fixed Paraffin-Embedded (FFPE) tissues. Patients with available data were included.

^c^ For one patient in the American cohort, it was unclear if that patient had stage III or stage IV colon cancer, therefore this patient was removed from analyses stratifying by TNM stage.

### MiR-34a, -34b and -34c are increased in colon cancer, and increased miR-34b/c is associated with advanced tumors and poor prognosis

**Fig 1:**
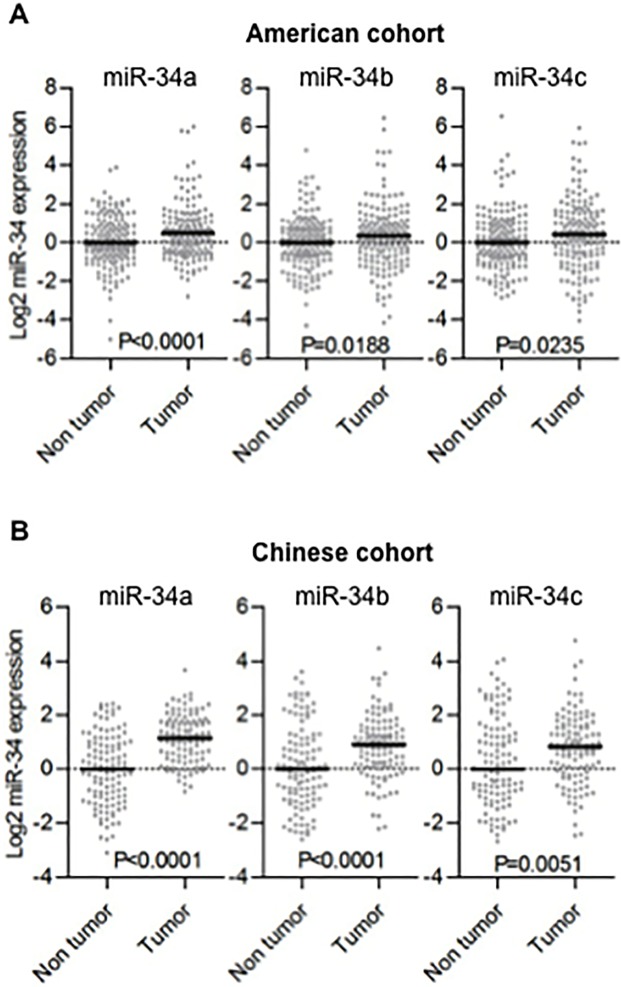
The miR-34 family members significantly increase in colon tumors compared to adjacent non-tumor tissues. (A, B) Dot plots represent miR-34a/b/c threshold cycle values from TaqMan qRT-PCR normalized to U66 in American cohort (A) and Chinese cohort (B). Horizontal bars indicate median expression value. Wilcoxon matched-pairs test was used. MiR-34a was not detectable in one patient and this patient was excluded from all analyses.

**Fig 2:**
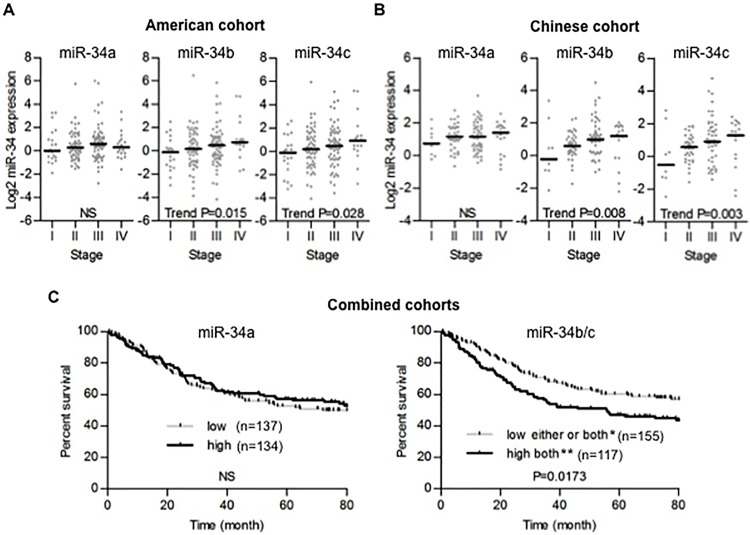
The high expression of miR-34b/c is associated with advanced colon tumor and poor prognosis. (A, B) Tissues were ordered from TNM stage I to IV tumors in the American cohort (A) and the Chinese cohort (B). Dot plots represent miR-34a/b/c threshold cycle values from TaqMan qRT-PCR normalized to U66. Horizontal bars indicate median expression value. The Cuzick nonparametric test for trend was used to evaluate trends. (C) Kaplan-Meier survival analysis of all stage cases in the American and Chinese combined cohorts stratified by median miR-34a expression and combined miR-34b/c expression. *Cases with low miR-34b and/or low miR-34c. **Cases with both high miR-34b and high miR-34c.

We performed TaqMan qRT-PCR of miR-34a, miR-34b and miR-34c in colon tumors and adjacent non-tumor tissues from both the American and the Chinese cohorts. Unexpectedly, The expression of these 3 miRNAs was significantly increased in tumors compared to non-tumor tissues in both cohorts (Fig 1A and 1B). These increases were relatively small, but highly significant. In the US cohort, the fold-change increase was 1.42, 1.27 and 1.25 for miR-34a, -34b and -34c respectively. In the Chinese cohorts, the fold change increase was 2.26, 1.64 and 1.40, respectively. There was no significant association between miR-34a expression and TNM staging (Fig 2A and 2B left panels), while increased miR-34b and -34c were associated with higher TNM staging in both cohorts (test for trend, P<0.05; Fig 2A middle and right panels, P<0.01; Fig 2B middle and right panels). MiR-34b and miR-34c are co-transcribed from a bicistronic transcript and their expression should be highly correlated [9]. In agreement with this, the expression of miR-34b and -34c were highly correlated in both tumors and non-tumor tissues from each cohort (Pearson’s correlation r>0.9, P<0.0001; S1A and S1B Fig). Thus, we used combined miR-34b/c expression into the following survival analysis. The American and the Chinese cohorts were combined to increase the statistical power for this analysis and expression levels for each miRNA were dichotomized based on median values. There was no significant association between miR-34a expression and prognosis (Fig 2C left panel). On the other hand, colon cancer patients with high expression of both miR-34b and -34c in tumor showed significant poor prognosis (log-rank P<0.05; Fig 2C right panel). When the survival analysis was performed on each cohort separately, the differences were not statistically significant (S2A and S2B Fig). In multivariate analysis, miR-34b/c was not significantly associated with survival when adjusted for TNM staging (data not shown).

### The association between miR-34 expression and TP53 mutation

**Fig 3:**
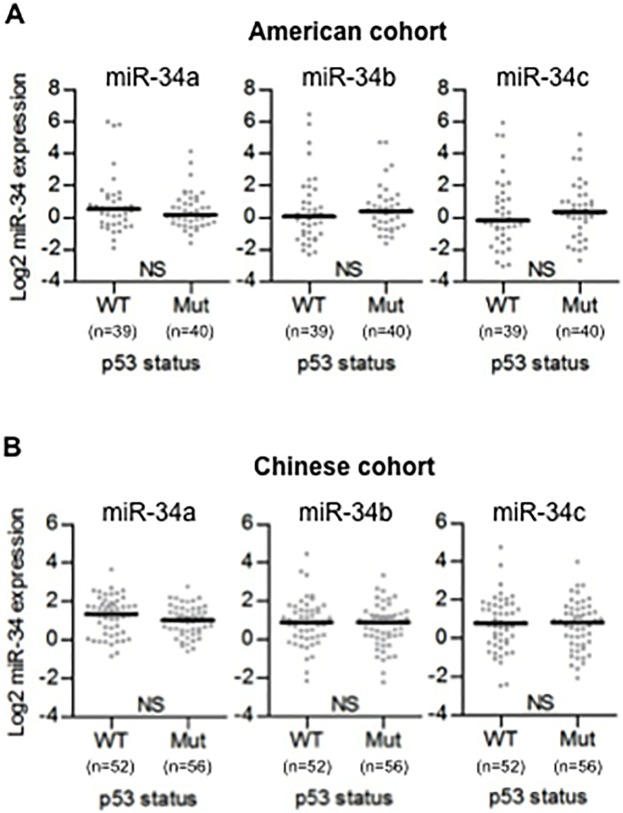
TP53 mutation is not associated with the expression of miR-34 family. (A, B) TP53 mutational status was evaluated by immunohistochemistry using Formalin-Fixed Paraffin-Embedded (FFPE) tissues. Dot plots represent miR-34a/b/c threshold cycle values of tumor tissue from TaqMan qRT-PCR normalized to U66 in American cohort (A) and Chinese cohort (B). Horizontal bars indicate median expression value. Mann-Whitney test was used. WT: wild-type p53, Mut: mutant p53.

Since miR-34a, miR-34b, and miR-34c were each systematically altered in colon tumors, we next attempted to determine possible reasons for this altered expression. Several studies indicate that p53 can bind to the promoter and activate the transcription of miR-34 family [10–12]. To address this, we examined whether miR-34 expression was associated with TP53 mutational status. TP53 mutational status can be examined either through immunohistochemistry or through direct sequencing. In the present study, the TP53 mutational status was assumed to be ‘wild-type’ or ‘mutant’ by immunohistochemical staining of p53 [22]. Contrary to our expectations, the expression of miR-34 family was not associated with TP53 mutational status in either the American and Chinese cohorts (Fig 3A and 3B). Additionally, TP53 mRNA expression was not associated with miR-34a (R = 0.005, p = 0.97) or miR-34b/c (R = 0.013, p = 0.92) expression based on microarray expression values of TP53 (S1 Table).

### Ingenuity Pathway Analysis (IPA) predicts transcription regulators that potentially associated with miR-34 up-regulation

In order to investigate potential reasons for the altered expression of the miR-34 family, we used Affymetrix microarray data to identify genes that correlated with miR-34a and -34b/c and examined these genes with Ingenuity Pathway Analysis (IPA). MiR-34b/c correlated with 649 genes in Affymetrix microarray data of 56 tumors from the American cohort (S1 Table; P<0.001), and IPA was used to examine this set of genes. Increased expression of miR-34b/c was associated with active transcriptional activity of TP53, SMAD3, CBL, SNAI1, HTT, TWIST1 and CTNNB1 while it was associated with reduced transcriptional activity of MYCN, AHR, SMAD7, MYC, SPDEF and PPARG (Table 2, S2 Table). Similar analysis on miR-34a expression identified 385 genes that were correlated with miR-34a (S1 Table; P<0.001). Increased expression of miR-34a was associated with increased activity of 48 transcriptional regulators including TP53 and decreased activity of 17 transcriptional regulators (S3 Table).

**Table 2:** List of Transcription Regulators that are potentially involved with miR-34b/c expression predicted by Ingenuity Pathway Analysis in 56 tumors.

Transcription Regulator	Activation State	z-score	p-value of overlap	Target molecules^a^
TP53	Activated	3.618	7.50E-07	64 genes
SMAD3	Activated	3.289	4.48E-05	18 genes
CBL	Activated	2.705	1.72E-03	5 genes
SNAI1	Activated	2.490	1.29E-04	7 genes
HTT	Activated	2.400	3.63E-05	43 genes
TWIST1	Activated	2.256	7.71E-03	5 genes
CTNNB1	Activated	2.160	1.82E-05	36 genes
MYCN	Inhibited	-4.242	4.69E-08	26 genes
AHR	Inhibited	-4.118	4.11E-12	36 genes
SMAD7	Inhibited	-3.698	3.43E-12	23 genes
MYC	Inhibited	-3.348	8.65E-05	43 genes
SPDEF	Inhibited	-3.290	1.05E-18	24 genes
PPARG	Inhibited	-2.210	4.23E-02	18 genes

^a^ All target molecules are listed in S3 Table.

### The comparison of miR-34 expression between colon epithelium and stroma with laser microdissection (LMD) technique

**Fig 4:**
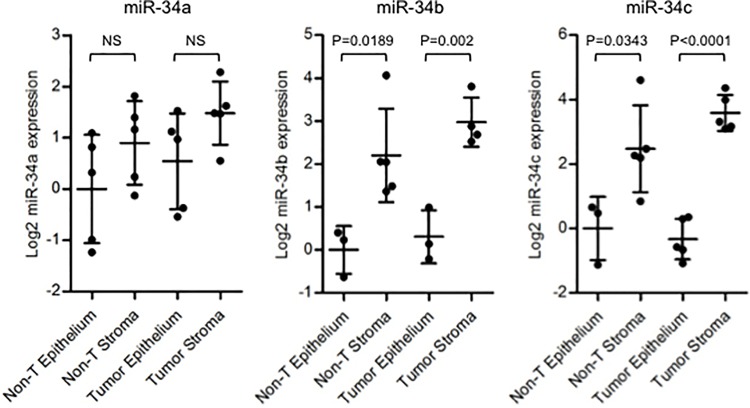
miR-34 family tends to be expressed predominantly in stromal tissue. RNA samples from cancer epithelium, cancer stroma, normal adjacent epithelium and normal adjacent stroma were extracted separately using laser microdissection technique from 5 paired samples in the American cohort. Dot plots represent miR-34a/b/c threshold cycle values of tumor tissue from TaqMan qRT-PCR normalized to U48. Horizontal bars indicate median expression value. Un-paired t test was used.

Altered expression of oncogenic miRNAs in colorectal cancer stromal tissue might be associated with cancer progression [23]. To examine where the miR-34 family is expressed in, we extracted RNA from cancer epithelium, cancer stroma, normal adjacent epithelium and normal adjacent stroma separately using laser microdissection for miR-34 expression analysis (S3 Fig). Five colon tumor and 5 adjacent non-tumor frozen tissue samples were obtained form the American cohort. MiR-34b and -34c expression levels in stroma were significantly higher as compared with that of epithelium in both cancer tissues and in normal adjacent tissues (Fig 4 middle and right panels). MiR-34a showed similar trend although the differences were not significant (Fig 4 left panel). We further examined the publicly available microarray dataset (GSE35602), in which miRNA expression profiles of micro-dissected colon cancer stromal and epithelial tissues were analyzed, demonstrating a clear trend of higher miR-34b and -34c expression in cancer stroma (S4 Fig). This microarray data are consistent with our own qRT-PCR analysis using LMD samples, indicating that miR-34b and -34c are expressed at higher levels in stomal tissues. In addition, we have performed H&E on 82 of the tissues from the American cohort to examine if there was an association with increased stromal area and any of the miR-34 family. There was no significant correlation between tumor/stroma ratio and expression levels of miR-34 (S5 Fig). These findings based on LMD analyses suggest that stromal cells serve the major source of miR-34b/c rather than epithelial cells. Hence, miR-34b/c expression detected in macro-dissected tumor samples (as shown in Fig 1) seems to mainly represent stromal expression of miR-34b/c.

## Discussion

In the present study, we demonstrated that: 1) miR-34a/b/c were increased in human colon cancers compared to adjacent non-tumor tissues. 2) Increased expression of miR-34b/c was observed in more advanced tumors and associated with poor prognosis. 3) The expression of miR-34 family was not associated with TP53 mutational status although pathway analysis showed association between miR-34 expression and TP53 transcriptional activity. 4) The expression of miR-34b and -34c were increased significantly in stromal tissues compared with epitheliums.

In colorectal cancer, prior studies have focused on a tumor suppressive function for miR-34 family as shown in many kinds of malignancy [9]. In vitro experiments have demonstrated that ectopic expression of miR-34a into human colon cancer cell lines induced apoptosis, senescence and inhibited migration and invasion by targeting E2F, SIRT1 and Fra-1 [24–26]. Considering the tumor suppressive function of miR-34 as shown previously, we had expected that miR-34 family was suppressed in human colon cancer and decreased expression was associated with poor prognosis when we started the present study. Previously, Roy et al. demonstrated that the expression of miR-34a and miR-34c were down-regulated in colon cancer specimens compared to adjacent colonic mucosa in small number expression analysis (n = 10) [27]. Contrary to our expectation, all miR-34 family members were increased significantly in colon cancer tissues compared with adjacent normal tissues, and increased miR-34b/c was associated with poor prognosis. As a matter of fact, the present data is consistent with several recent reports analyzing miR-34 expression and its prognostic value. MiR-34 family members have been found to increase in colorectal cancer [28], gastric cancer [29], renal cell carcinoma [30, 31], lung squamous cell carcinoma [32] and pediatric leukemia [33]. In addition, Wang et al. demonstrated that the miR-34a expression was higher in rectal cancer having more advanced TNM stage [28], and Svoboda et al. demonstrated that miR-34b up-regulation was associated with poor prognosis in triple-negative (ER-, PgR-, HER2-) breast cancer patients [34]. The fact that miR-34 family is up-regulated in human cancer and its up-regulation is associated with poor prognosis, seems to be conflicted with tumor suppressive role of miR-34 demonstrated in previous reports.

On the basis of these findings, we analyzed epithelial and stromal expression of miR-34 separately using laser microdissection technique to examine if cancer cells express miR-34. Interestingly, all miR-34 family members, especially miR-34b and -34c, were increased in stromal tissues compared with epithelium. In addition, the expression levels of miR-34 seemed to be the highest in cancer stromal tissues. These findings suggest that colon cancer stroma is mainly responsible for increased miR-34b/c expression, which were associated with advanced TNM stage and poor prognosis. However, the reason for the up-regulation of miR-34 in stromal tissue is still unclear. Nishida et al. demonstrated that several miRNAs were significantly altered in cancer stroma, suggesting an important role of stromal miRNAs in cancer progression [23]. Stromal tissues consist of inflammatory cells, immunocompetent cells, endothelial cells and fibroblasts. One possible explanation of our findings is that miR-34 up-regulation in stromal tissues might reflect inflammation of tumor tissues, as it is well known that inflammation contributes to the development of cancer, including colon cancer [35]. Mathe et al. demonstrated that mice treated with C. parvum, to induce inflammation, showed significant increase of miR-21, miR-29b and miR-34a/b/c expression in the spleen [36]. Taken together, the fact that miR-34b/c was increased in cancer stromal tissues, especially in more advanced colon cancer, might be associated with inflammation contributing to cancer progression. In a recent study, Bu et al. have demonstrated that miR-34a regulated bimodal switch targets Notch in colon cancer stem cells [37]. The finding may demonstrate one more possibility that different level of miR-34 expression could be significant for miR-34-mediated interaction between human colon cancer and stroma.

In the present study, we demonstrated conflicting findings. As shown in Fig 3, the expression of miR-34 family was not associated with TP53 mutational status. However, Ingenuity Pathway Analysis demonstrated that Increased expression of miR-34a/b/c was associated with active transcriptional activity of TP53 (Table 2, S2 Table, S3 Table).

Although it has been well known that miR-34 was direct target of p53, a recent study demonstrated that mouse models that contain deletions for miR-34a, miR-34b and miR-34c still retain p53 function and the expression of these microRNAs is largely independent of p53 status [38]. In general, cancer stromal tissues are considered to harbor wild-type p53 regardless of p53 mutational status of cancer tissues. Considering the fact that miR-34 was expressed in stromal tissues predominantly, miR-34 detected in macro-dissected cancer tissues seems to be mainly regulated by cancer stromal tissues harboring wild-type p53. That may be a plausible explanation that the expression of miR-34 family was not associated with TP53 mutational status of cancer.

## Conclusions

In conclusion, we showed novel aspects of miR-34 family in human colon cancer. The demonstrated findings imply that increased miR-34 in stromal tissues may have important roles in colon cancer progression. Further analysis is needed to clarify the function and miR-34-mediated interaction between human colon cancer and stroma.

## Supporting Information

S1 Fig The expression of miR-34b and -34c strictly parallels. Correlation analysis of miR-34b/c expression in tumor and non-tumor tissue was performed. A, American cohort. B, Chinese cohort. Dot plots represent miR-34b/c threshold cycle values from TaqMan qRT-PCR normalized to U66. Pearson’s correlation test was used.
(TIFF)

S2 Fig Correlation between miR-34 expression and survival. Kaplan-Meier survival analysis of all stage cases in the American cohort (A) and Chinese cohort (B) stratified by median miR-34a expression and combined miR-34b/c expression. *Cases with low miR-34b and/or low miR-34c. **Cases with both high miR-34b and high miR-34c.
(TIFF)

S3 Fig Laser microdissection technique. RNA samples from cancer epithelium, cancer stroma, normal adjacent epithelium and normal adjacent stroma were extracted separately using laser microdissection technique from 5 colon tumors and 5 adjacent non-tumor tissues obtained from American cohort.
(TIFF)

S4 Fig The miR-34 family members increase in stroma compared to epithelium. Dot plots represent miR-34a/b/c threshold cycle values from TaqMan qRT-PCR. Horizontal bars indicate median expression value.
(TIFF)

S5 Fig The miR-34 family does not associated with increased stromal area. The H&E staining on 82 of the tumor tissues from the American cohort was performed, and the area ratio of tumor and stroma (TS-ratio) was determined. Dot plots represent miR-34a/b/c threshold cycle values from TaqMan qRT-PCR and TS-ratio. Pearson’s correlation test was used.
(TIFF)

S1 Table Correlation analysis between Affymetrix microarray data and miR-34 family expression in 56 colon tumors. (XLSX)

S2 Table List of Transcription Regulators that are potentially involved with miR-34b/c expression predicted by Ingenuity Pathway Analysis in 56 colon tumors. (XLSX)

S3 Table List of Transcription Regulators that are potentially involved with miR-34a expression predicted by Ingenuity Pathway Analysis in 56 colon tumors. (XLSX)
