# Supplementary figures and images for: Increased MicroRNA-34b and -34c Predominantly Expressed in Stromal Tissues Is Associated with Poor Prognosis in Human Colon Cancer
Source: PLoS One. 2015 Apr 20;10(4):e0124899. doi: 10.1371/journal.pone.0124899 (PMC4404052; doi:10.1371/journal.pone.0124899)

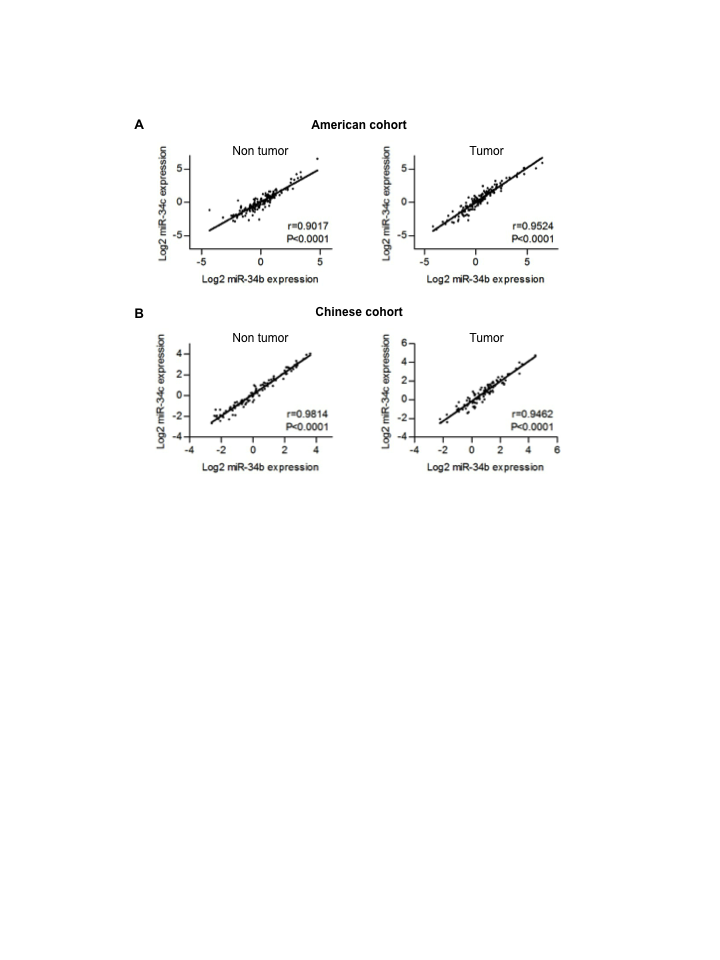

Supplement: S1 Fig — Correlation analysis of miR-34b/c expression in tumor and non-tumor tissue was performed. A, American cohort. B, Chinese cohort. Dot plots represent miR-34b/c threshold cycle values from TaqMan qRT-PCR normalized to U66. Pearson’s correlation test was used. (TIFF) [file pone.0124899.s001.tiff]

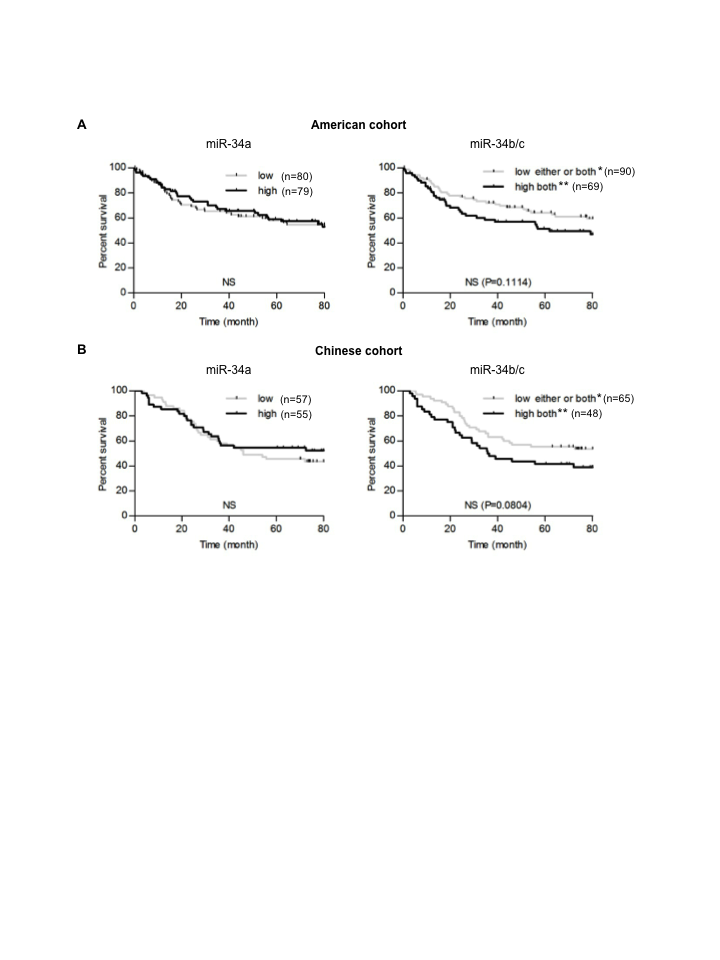

Supplement: S2 Fig — Kaplan-Meier survival analysis of all stage cases in the American cohort (A) and Chinese cohort (B) stratified by median miR-34a expression and combined miR-34b/c expression. *Cases with low miR-34b and/or low miR-34c. **Cases with both high miR-34b and high miR-34c. (TIFF) [file pone.0124899.s002.tiff]

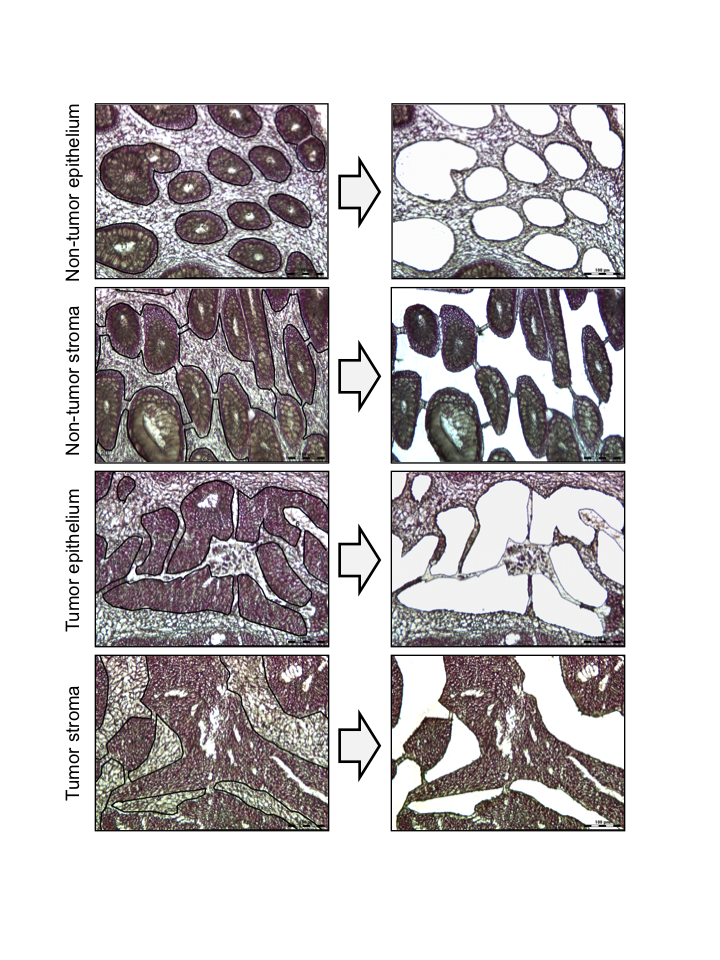

Supplement: S3 Fig — RNA samples from cancer epithelium, cancer stroma, normal adjacent epithelium and normal adjacent stroma were extracted separately using laser microdissection technique from 5 colon tumors and 5 adjacent non-tumor tissues obtained from American cohort. (TIFF) [file pone.0124899.s003.tiff]

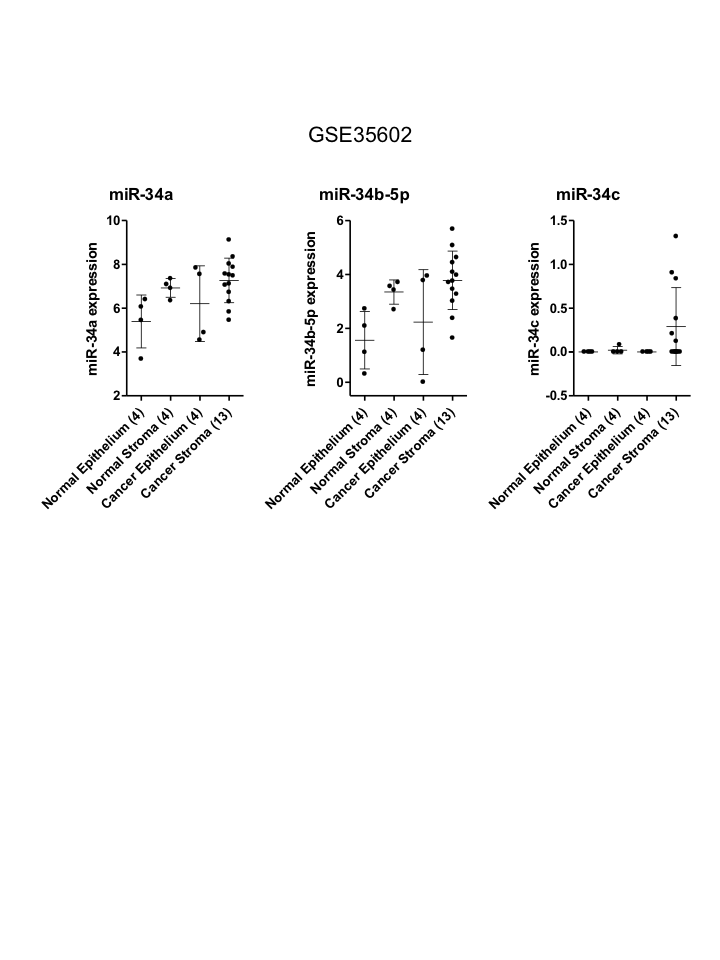

Supplement: S4 Fig — Dot plots represent miR-34a/b/c threshold cycle values from TaqMan qRT-PCR. Horizontal bars indicate median expression value. (TIFF) [file pone.0124899.s004.tiff]

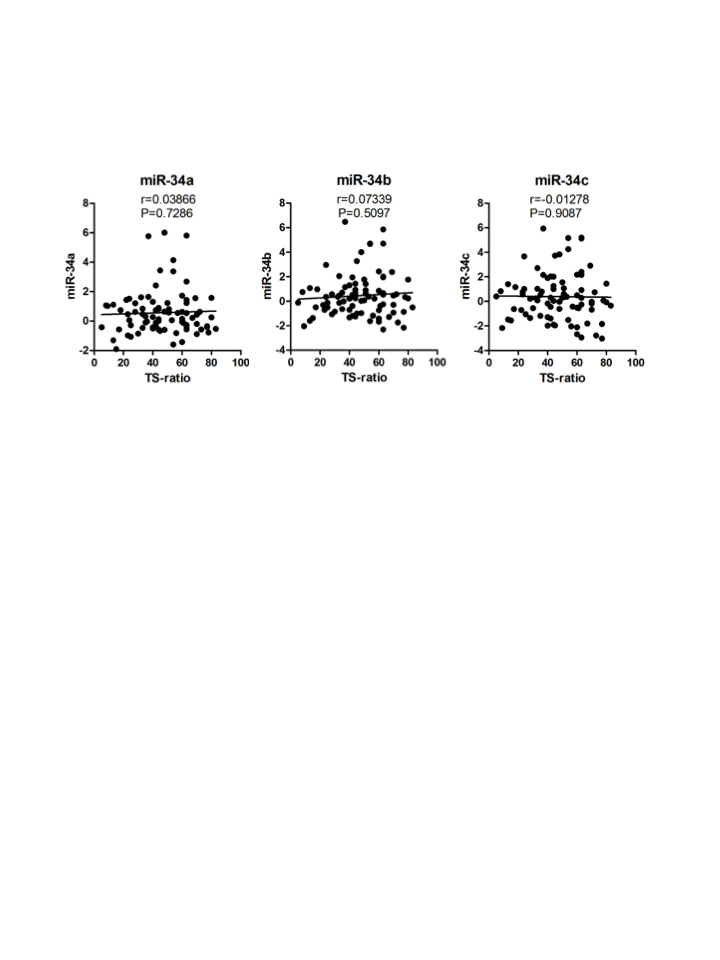

Supplement: S5 Fig — The H&E staining on 82 of the tumor tissues from the American cohort was performed, and the area ratio of tumor and stroma (TS-ratio) was determined. Dot plots represent miR-34a/b/c threshold cycle values from TaqMan qRT-PCR and TS-ratio. Pearson’s correlation test was used. (TIFF) [file pone.0124899.s005.tiff]
